# Heat Stress Decreases Intestinal Physiological Function and Facilitates the Proliferation of Harmful Intestinal Microbiota in Sturgeons

**DOI:** 10.3389/fmicb.2022.755369

**Published:** 2022-03-07

**Authors:** Shiyong Yang, Chaoyang Zhang, Wenqiang Xu, Datian Li, Yang Feng, Jiayun Wu, Wei Luo, Xiaogang Du, Zongjun Du, Xiaoli Huang

**Affiliations:** ^1^Department of Aquaculture, Sichuan Agricultural University, Chengdu, China; ^2^Basic Veterinary Science, Sichuan Agricultural University, Chengdu, China; ^3^College of Life Sciences, Sichuan Agricultural University, Ya’an, China

**Keywords:** heat stress, sturgeon, valve intestine, microbiota disorder, physiological dysfunction

## Abstract

Heat is a common source of stress in aquatic environments and can alter the physiological and metabolic functions of aquatic animals, especially their intestinal function. Here, the effects of heat stress on the structure and function of the intestine and the characteristics of the intestinal microbiota were studied in sturgeon (*Acipenser baerii* ♀ × *Acipenser schrenckii* ♂ hybrid F1). Sturgeons were exposed to sub-extreme (24°C) and extreme (28°C) high water temperatures for 12 days. The heat stress caused systemic damage to the intestine of sturgeons, which displayed severe enteritis in the valve intestine. The microbial diversity analysis showed that heat stress led to the disorder in intestinal microbiota, manifesting as an explosive increase in the abundance of thermophilic intestinal pathogens such as *Plesiomonas*, *Cetobacterium*, and *Aeromonas* and causing physiological dysfunction in the sturgeons. The disorder was followed by significant inhibition of intestinal digestion with reduced chymotrypsin, α-amylase, and lipase activities in the valve intestine and of antioxidant function with reduced peroxidase (POD) and catalase (CAT) activities. Simultaneously, heat stress reduced the thermal tolerance of sturgeons by reducing *Grp75* expression and damaged the valve intestine’s repair ability with increased *Tgf-*β expression. The results confirmed that heat stress damaged the sturgeon intestines obviously and disturbed the intestinal microbiota, resulting in serious physiological dysfunction. The present study investigated the mechanism of the effect of heat stress on the sturgeon intestine and will help develop strategies to improve the resistance to thermal stress for wild and cultured sturgeons.

## Introduction

**FIGURE 1:**
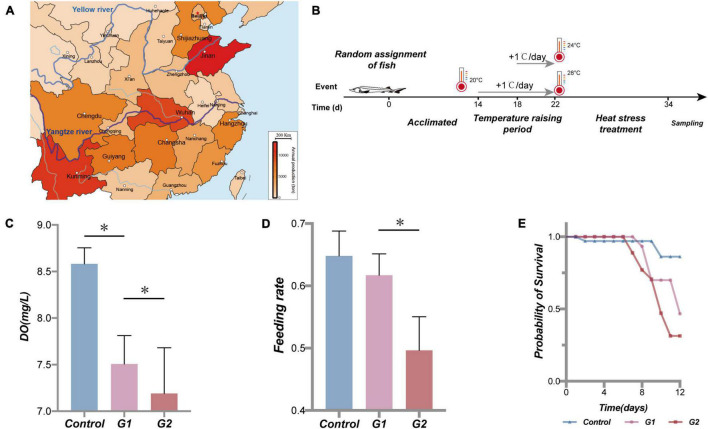
Sturgeon production in part of the province in China in 2017 (Le-jun and Yong-hui, 2018), water temperature scheme applied to the sturgeon in the present study, effects of heat stress on water dissolved oxygen, sturgeon feeding rate, and cumulative survival rate. Sturgeon production in 2017 **(A)**. During the experiment, each tank volume was 0.5 m^3^, dissolved oxygen was maintained above 7.5 mg/L, and pH was 7.4 ± 0.4. Feed was added twice a day (at a 7-h interval); the daily feed amount was 2% of the fish body mass. The water in the tanks was pretreated with UV light and an aeration process, and 25% of the culture water was renewed every day. Fish were held in a 12-h:12-h light/dark cycle **(B)**. **(C)** Dissolved oxygen (DO) in different treatments. **(D)** Feeding rate of sturgeon in different groups. **(E)** Fraction survival (%) of sturgeon during heat exposure. (*) Represents a significant difference between groups (*p* < 0.05).

Heat stress in local waters is likely to worsen due to global warming, threatening aquatic animals and potentially altering their behavior, growth, development, reproduction, and digestion (Hui-Huang et al., 2012; Miller et al., 2015; Aidos et al., 2020). Heat stress has severe consequences including death to fish (Yang et al., 2021). Heat stress threatens the survival of cold-water fishes, especially for sturgeons whose wellbeing closely relates to the environmental temperature. Zhang H. et al. (2019) found that the water temperature in the Yangtze River basin, which is the primary habitat of Chinese sturgeons, has increased by 1 to 3.5°C in autumn and 2 to 5°C in winter over the past 60 years (Zhang H. et al., 2019). Similarly, Larnier et al. (2010) found that the average surface water temperature in summer in Garonne, where the European sturgeon (*Acipenser sturio*) mainly lives, rose by 2.9°C between 1978 and 2005 (Larnier et al., 2010). Temperature increases not only directly delay the spawning of sturgeons but also can lead to degeneration in gonad development (Zhang H. et al., 2019). Affected sturgeons have been observed to have problems related to feeding (Mizanur et al., 2014), growth (Aidos et al., 2020), and reproduction, and in some cases, increased mortality has occurred (Lankford et al., 2003; Ficke et al., 2007; Hassell et al., 2008). The hybrid sturgeon (*Acipenser baerii* ♀ × *Acipenser schrenckii* ♂ hybrid F1) is a kind of commercial sturgeon valued globally for its flesh (Bronzi et al., 2019). Sturgeon habitats in China have historically been concentrated in the Yangtze and Yellow Rivers, but the temperatures of these rivers are rising (Liu et al., 2009; Zhang H. et al., 2019; Figure 1A). The abnormally high water temperatures from July to September present an especially difficult challenge for this cold-water fish; and numerous studies have associated immune decline, illness, and death with rising river temperatures (Shen et al., 2014; Castellano et al., 2017; Zhang H. et al., 2019). Therefore, the present study investigated the potential mechanisms by which heat stress influences the yields of commercial sturgeons.

The intestine is the largest organ involved in the digestion and absorption of nutrients, as well as the largest organ involved in the immunity, defense, and endocrine systems in fish (Zhu et al., 2013). In freshwater fish, digestive enzyme activities in the intestinal mucosa of *Carassius auratus*, *Cyprinus carpio*, *Rutilus*, and *Perca fluviatilis* decreased under high water temperatures, and rapid increases in water temperature adversely affected the rate of carbohydrate hydrolysis and lowered the thermal tolerance of the intestinal tract (Golovanova et al., 2013). In addition, high water temperatures reduced the secretion of digestive enzymes in fish intestines, resulting in decreased intestinal chyme transport time and digestibility (Miegel et al., 2010; Tirsgaard et al., 2015). Stable gastrointestinal function contributes to the normal biological function of sturgeons and improves the yield and quality of the meat of commercial sturgeons, but the effects of heat stress on the structure and function of the sturgeon intestine are still unknown.

Intestinal microbiota in fish have dynamic compositions of aerobic bacteria, facultative anaerobes, and anaerobic bacteria (Evariste et al., 2019). The ecological balance of intestinal microbiota is important for maintaining normal feeding behavior, growth performance, digestive capability, and fecundity in fish (Kunz et al., 2009; Desai et al., 2016; Chenggang and Wenjing, 2017). Studies have shown that increased temperatures can disrupt the balance of intestinal microbiota and facilitate the growth of pathogenic bacteria. High water temperatures have been shown to trigger explosions of the pathogen *Sphingomonas* that can decimate sea cucumber populations (Zi-Jiao et al., 2019). Larios Soriano et al. (2018) confirmed that increased water temperatures resulted in increased abundance and richness of intestinal microbiota in the yellowtail kingfish *Seriola lalandi*, along with an increased abundance of a large number of harmful bacteria (Larios Soriano et al., 2018). A study of lake sturgeon (*Acipenser fulvescens*) found a dynamic relationship between the composition of intestinal microbiota and the physiology of the host gastrointestinal tract (Razak and Scribner, 2020). However, the effects of high water temperatures on the intestinal microbiota of cold-water fish like sturgeons remain to be studied.

This study investigated the composition of intestinal microbiota and physiological and biochemical indexes in the intestine of sturgeons under different water temperatures. It revealed the mechanism of the effect of heat stress on sturgeon intestines, and the results will provide guidance toward managing thermal stress in wild and cultured sturgeons.

## Materials and Methods

### Fish Maintenance and Treatment Protocols

All animal handling procedures were approved by the Animal Care and Use Committee of Sichuan Agricultural University in accordance with the Animal Experiment Guidelines of Sichuan Agricultural University, license no. ZCY-2019202031. For the experiment, a total of 180 6-month-old healthy juvenile sturgeons (*A. baerii* ♀ × *A. schrenckii* ♂ hybrid F1) of 76.37 ± 9.45 g and 23.54 ± 2.41 cm length were purchased from Sichuan Runzhao Fishery Co., Ltd., Sichuan, China.

Referring to the study of Mai et al. (2014), Bai et al. (2012), and the data collected at the farm, 18 to 20°C was deemed to be the optimal temperature range for sturgeon growth (Bai et al., 2012; Mai et al., 2014). The fish were acclimated in the laboratory at 20°C for 2 weeks prior to the experiment. The sturgeons were then randomly divided into three groups, a control group kept at 20°C and two elevated temperature groups at 24°C (G1) and 28°C (G2) (Yang et al., 2021). Each group included four parallel tanks with 15 fish each. The experimental heating and heat treatment steps are illustrated in Figure 1B. The control group was held at 20°C at room temperature for the duration of the experiment. The G1 and G2 groups were also held in room temperature water for the first 14 days, and then the temperature was increased by 1°C/day until reaching the experimental temperatures of 24 and 28°C on day 22 where the temperature was maintained until day 34. During the treatment period, the state of the experimental fish was continuously observed for 24 h. Oxygen was continuously supplied by the oxygen pumps for 24 h, and dead or dying fish were quickly removed.

During the experiment, the feeding rates and deaths of the sturgeons in each group were recorded daily, along with dissolved oxygen in the water. On day 34, upon completion of the heat treatment, the sturgeons in each group were euthanized with tricaine mesylate (MS-222) (Sigma-Aldrich, Beijing, China). Blood was collected *via* the caudal vein, and fish were dissected for biochemical examination. The histopathology, ultrastructure, enzyme activity, mRNA expression, and intestinal microbiota of gastrointestinal tissues and contents were examined.

### Serum Biochemical Analysis

The blood was centrifuged at 3,500 × *g* at 4°C for 10 min. The serum of three fish was selected in each group randomly to form a pooled sample. Serum samples from each group (*n* = 3) were measured for albumin (ALB), alkaline phosphatase (ALKP), alanine transaminase (ALT), amylase (AMYL), blood urea (UREA), calcium (CA), cholesterol (CHOL), creatinine (CREA), gamma-glutamyl transpeptidase (GGT), globulin (GLOB), glucose (GLU), lipase (LPS), phosphatase (PHOS), total bilirubin (TBIL), and total protein (TP). All these indicators were measured using the IDEXX Catalyst One biochemical analyzer (IDEXX, Catalyst One, Westbrook, ME, United States).

### Examination of Anatomy, Histopathology, and Ultrastructure

The sturgeons from day 35 were euthanized and dissected for examination. The physical morphology of the gastrointestinal tract was observed. Tissue specimens of the intestines were fixed in 4% polyformaldehyde solution and, following a routine process, fixed in paraffin and stained with H&E. Histological slides were examined under a light microscope (Nikon, Tokyo, Japan). Intestinal tissue was placed in a fixative of 2.5% glutaraldehyde in pH 7.4 cacodylate buffer, washed three times in phosphate-buffered saline (PBS), and post-fixed with 1% osmium tetroxide. After dehydration in graded alcohol, the tissues were embedded in Araldite. The blocks were sectioned on a microtome with a glass knife. The 6.575-μm-thick sections were placed on uncoated copper grids, stained with uranyl acetate, and post-stained with 0.2% lead citrate. A transmission electron microscope (HT7700, Hitachi, Tokyo, Japan) was used to observe and collect images for analysis.

The degrees of hemorrhage, edema, deposits, hypertrophy, hyperplasia, atrophy, infiltration, and necrosis of the gastrointestinal tract were evaluated according to Huang et al. (2019). Every change was given an *S* score ranging from 0 to 6, depending on the degree and extent of the change: unchanged (0), mild change (2), moderate change (4), and severe change (6) as a diffuse lesion. Intermediate values were also considered. The organ index (*I* = Σ_*t*_Σ_*alt*_ [*S* × ω_*IF*_]) and total index (*I* = Σ_*Org*_Σ_*t*_Σ_*alt*_ [S × ω_*IF*_]) were calculated for each experimental group in this study, where ω_*IF*_ was the important factor.

### Antioxidant Index and Digestive Enzyme Activity Determination

The antioxidant and digestive enzyme activity indexes for the intestine and serum were determined using diagnostic kits produced by Nan Jing Jian Cheng Bioengineering Institute (Nanjing, China) following the manufacturer’s instructions. Approximately 0.1-g valve intestinal tissue was homogenized with 0.9 ml of 0.65% NaCl solution in a homogenizer on ice. The homogenates and whole blood were then centrifuged at 3,500 × *g* 10 min at 4°C, and total protein in the supernatant of the tissue was determined with a protein quantification kit (A045-2). The diagnostic kits used in this experiment were for catalase (CAT) (A007-1), glutathione peroxidase (GSH-Px) (A005), peroxidase (POD) (A084-1), alpha amylase (α-AMS) (C016-1), LPS (A054-1), and chymotrypsin (CHY) (A080-3).

### Real-Time Quantitative Polymerase Chain Reaction

Primer sequences for β*-actin*, *ef1a*, *Hsp60*, *Hsp70*, *Tgf-*β, and *Grp75* were designed and synthesized based on unpublished transcriptome data by the Beijing Qingke Biotech Co., Ltd. (Beijing, China). The housekeeping genes β*-actin* and *ef1a* were used as internal references. All primer sequences are shown in Table 1. Total RNA was extracted from frozen intestine samples using an animal total RNA isolation kit (Foregene, Chengdu, China) according to the manufacturer’s instructions. The integrity and quality of the RNA were assessed using 1% agarose gel electrophoresis. The RNA concentration and purity were spectrophotometrically determined at 260/280 nm. Complementary DNA (cDNA) was synthesized using the RR047A kit (TaKaRa, Dalian, China) according to the manufacturer’s instructions. The polymerase chain reaction (PCR) was performed using a CFX96 (Bio-Rad, Hercules, CA, United States) according to the manufacturer’s instructions. Reactions were performed in a 10-μl mixture made up of 1 μl of diluted cDNA, 5 μl of SYBR green master, 0.5 μl of forward primer, 0.5 μl of reverse primer, and 3 μl of PCR-grade water. The PCR program was 95°C for 1 min and 40 repeated cycles of 95°C for 10 s, and the appropriate melting temperature was 30 s. The melting curve revealed a single peak for each PCR product. Relative mRNA expression was calculated using the 2^–ΔΔ*Ct*^ method with the following formula: *F* = 2^–ΔΔ*Ct*^, ΔΔCt = (C_t, target gene_ − C_t, reference gene_) − (C_t, target gene_ − C_t, reference gene_) control.

**TABLE 1:** Primers of various genes detected with qPCR.

Symbol	Genes	Tm (°C)	Primer sequences (from 5′ to 3′)	Size (bp)
β-*actin*	β-*actin*	59.4	F:TGGACGCCCAAGACATCAGG R:GGTGACAATGCCGTGCTCG	127
*ef1a*	*ef1a*	59.4	F:TGAAGGCTGGCATGATCGTC R:AGGGTCTCGTGGTGCATTTC	118
*Hsp60*	*heat shock protein 60*	53.4	F:AGTTCCAGGACGCTTATCTG R:GTTCTGGTTTGCGATTTCCA	89
*Hsp70*	*heat shock protein 70*	53.7	F:GCCAGCGGTGGATTTCACT R:TGCTATTGCTTATGGCTTGGAC	136
*Tgf*-β	*transforming growth factor*-β	57.2	F:GCAGCTGTTCTTCAACATGT R:GTGCCCTTGTACAGCTCTAT	141
*Grp75*	*glucose regulated protein 75*	53.9	F:ACGGACTGAGTCAAGATGTC R:CTGTTTGCCTTCCATCACTG	131

### High-Throughput Sequencing of the Intestinal Microbiota

#### DNA Extraction and Purification

Fifteen individuals of hybrid F1 from each group of four batches were randomly selected and humanely euthanized with MS-222. Intestinal fecal matter from five sturgeons was combined to make a pooled sample with three pooled samples per group. To ensure the efficiency of DNA extraction, 0.5 g of feces in each pooled sample was considered necessary. All intestinal content samples were sent to Shanghai Majorbio Biopharm Technology Co., Ltd. (Shanghai, China) for genomic DNA extraction. The DNA was extracted from the intestinal feces using the Bacterial DNA Isolation Kit (DE-05311, Foregene Company, Limited, China) and Plant DNA Isolation Kit (DE-06111, Foregene Company, Limited, China), according to the manufacturer’s instructions. DNA concentration and quality were checked using a NanoDrop2000 (Thermo Fisher, Scotts Valley, CA, United States). The genomic DNA quality was assessed by 1% agarose gel electrophoresis. The V3–V4 region of the 16S rRNA gene was amplified by PCR with the primers proposed by Mori et al. (2014). Bacterial primer information: forward primer 338F: 5′-ACTCCTACGGGAGGCAGCAG-3′ and reverse primer 806R: 5′-GGACTACHVGGGTWTCTAAT-3′. The ITS1 (internal transcribed spacer) region of the fungi rRNA gene was amplified by PCR with the primers proposed by Adams et al. (2013). Fungal primer information: forward primer ITS1F: 5′-CTTGGTCATTTAGAGGAAGTAA-3′ and reverse primer ITS2R: 5′-GCTGCGTTCTTCATCGATGC-3′. The PCR products were detected by 2% agarose gel electrophoresis and purified with the AxyPrep DNA Gel Extraction Kit (Axygen, Corning, NY, United States), quantified using the QuantiFluorTM-ST Blue Fluorescence System (Promega, Beijing, China), and subjected to next-generation sequencing.

#### Sequencing and Quality Control

Sequencing of the 16S and ITS rDNA was performed on an Illumina Miseq PE300 platform (Illumina, San Diego, CA, United States) by Meiji Bioinformatics Technology Co., Ltd. (Shanghai, China). Two libraries were constructed for the V3–V4 and ITS1 amplicons. The paired-end (PE) sequencing was performed on the MiSeq system. The sequencing data were uploaded to the Sequence Read Archive at the National Center for Biotechnology Information (accession numbers PRJNA739968 and PRJNA738812). Based on the overlapping PE reads, pair-end double-ended sequences were merged into single sequences using Flash software (Version 1.2.11). Raw fastq files were demultiplexed and quality-filtered by removing those that were shorter than 50 bp and greater than 10 bp in libraries or had ambiguous nucleotides that constituted over 20% of the sequence. According to the overlap of PE reads, the PE reads were merged into a sequence, and the minimum overlap length was 10 bp. The maximum allowable mismatch ratio in the overlapping region of the spliced sequences was 0.2, and any unmatched sequences were screened. Samples were distinguished according to the barcodes and primers at the beginning and end of their sequences, and the sequence direction was adjusted. The allowed number of mismatches in the barcode was zero, and the maximum allowable number of primer mismatches was 2.

Non-repetitive sequences were extracted from the optimized sequences to reduce the number of redundant calculations in the middle of analysis.^1^ All single sequences without repetitions were removed.^2^ Operational taxonomic unit (OTU) clustering of non-repetitive sequences excluding single sequences was conducted according to 97% similarity. Chimeras were removed in the clustering process, and representative OTU sequences were obtained. All optimized sequences were mapped to the OTU representative sequences, and sequences that shared more than 97% similarity with the representative sequence were used to generate the OTU table. To obtain the species classification information corresponding to each OTU, the RDP classifier Bayesian algorithm was used to perform taxonomic analysis on the 97% similar level of OTU representative sequences. Each OTU was compared with the 16S rRNA database (Silva) and ITS rRNA database (UNITE) using BLAST analysis to obtain species classification information. In all sample analyses, species with relative abundance rates <0.01 were classified as “others.”

#### Intestinal Microbiota Analysis

Species observed (Sobs) were used to evaluate actual richness, the Shannon and Simpson indexes were used to assess microbiota diversity, the Ace and Chao indexes were used to assess microbiota richness. The Venn diagram was prepared based on the alpha diversity analysis performed in Mothur.^3^ The R language (version 3.3.1) was used to make the microbiota. Bar diagrams were used to visualize the dominant genus of each sample at the taxonomic level and the relative abundance (proportion) of each dominant genus in the sample. A beta diversity analysis was used to compare species diversity between different temperature groups. The abundances of the genus and the number of genera in each sample were counted. A visual circle diagram was used to reflect the connections among samples and the genus. The top 50 most abundant genera were used to visually study the microbiota composition using the heat map visualization method.

### Statistical Analysis

All data were shown as the mean ± SD and assessed for homogeneity of variance. The IBM SPSS version 27.0 Statistics software (IBM Corp., Armonk, NY, United States) was used for the statistical analyses. Indicators related to heat stress were analyzed using one-way ANOVAs, and *t*-tests and differences among the two temperature groups at the same time point as well as the differences within groups at different time points were analyzed. Duncan’s multiple comparison tests were used to compare means. The microbial diversity analysis database and software are shown in Supplementary Table 1. A normality test was conducted before the evaluation of the variables between the groups of the microbiota. The *p*-value was corrected by multiple hypothesis tests using the BH method (screening threshold: false discovery rate (FDR) <0.1, FDR = E(V/R), Abs (log_2_ fold change) ≥2). *p* < 0.05 and *p* < 0.01 were considered significant and extremely significant, respectively.

## Results

### The Effects of Heat Stress on Sturgeon Feeding, Survival Rate, and Physiological Indicators

As the water temperature increased, the dissolved oxygen in the tank significantly decreased in the three temperature groups (Figure 1C). Compared with the control and G1 groups, the fish in the G2 group had a significantly lower feeding rate (Figure 1D). The cumulative survival rate of the control group was significantly higher than that of both treatment groups, and it was higher in G1 than G2 (Figure 1E). The final survival rates of the control group were 86.2%, G1 group 46.7%, and G2 group 31.4% survival. Plasma biochemical indexes have been widely used to assess the physiological function in animals (Lei and Xiu-Mei, 2004). In this study, the ALB, ALKP, CHOL, GLOB, GLU, LIPA, PHOS, and TP were all higher in the highest temperature group. The LPS, ALKP, and PHOS can be used to specifically assess the physiological function of the digestive system, and their plasma levels increased significantly with increasing water temperature (Table 2). Overall, there was a significant decrease in feeding rate at the higher temperature, and it was speculated that the sturgeons’ digestion was impaired.

**TABLE 2:** Effects of heat stress on biochemical indicators in the serum of sturgeon.

Symbol	Item	Control	G1	G2
ALB (g/L)	Albumin	7.00 ± 0.82	8.00 ± 0.00	8.67 ± 1.25
ALKP (U/L)	Alkaline phosphatase	85.50 ± 11.50	121.00 ± 26.00	157.50 ± 9.50
AL (U/L)	Alanine transaminase	128.50 ± 27.50	175.67 ± 33.81	150.00 ± 44.97
AMYL (U/L)	Alpha amylase	5.00 ± 0.00	5.00 ± 0.00	5.00 ± 0.00
UREA (mmol/L)	Urea	0.63 ± 0.05	0.67 ± 0.05	0.67 ± 0.05
CA (mmol/L)	Creatine kinase	1.77 ± 0.05	1.87 ± 0.04	1.84 ± 0.03
CHOL (mmol/L)	Cholesterol	0.50 ± 0.27^a^	0.62 ± 0.04^a^	1.57 ± 0.02^b^
CREA (μmol/L)	Creatinine	25.00 ± 1.00^a^	13.33 ± 3.30^b^	28.00 ± 2.00^a^
GGT (U/L)	Gamma-glutamyl transpeptidase	0.00 ± 0.00	0.00 ± 0.00	0.00 ± 0.00
GLOB (g/L)	Globulin	10.00 ± 0.00^ab^	9.67 ± 1.70^a^	13.33 ± 1.70^b^
GLU (mmol/L)	Glucose	3.68 ± 1.45	4.44 ± 1.96	5.34 ± 0.56
LPS (U/L)	Lipase	34.00 ± 12.68^a^	67.00 ± 6.48^b^	77.00 ± 5.00^b^
PHOS (mmol/L)	Phosphatase	3.12 ± 0.42^a^	4.08 ± 0.48^ab^	4.34 ± 0.61^b^
TBIL (μmol/L)	Total bilirubin	2.00 ± 0.00	2.00 ± 0.00	2.00 ± 0.00
TP (g/L)	Total protein	17.00 ± 0.82^a^	17.67 ± 1.70^ab^	22.00 ± 2.83^b^

*Superscript a and b denote statistically significant differences among groups (p < 0.05).*

### Heat Stress Causes Enteritis of Valve Intestine in Sturgeons

**FIGURE 2:**
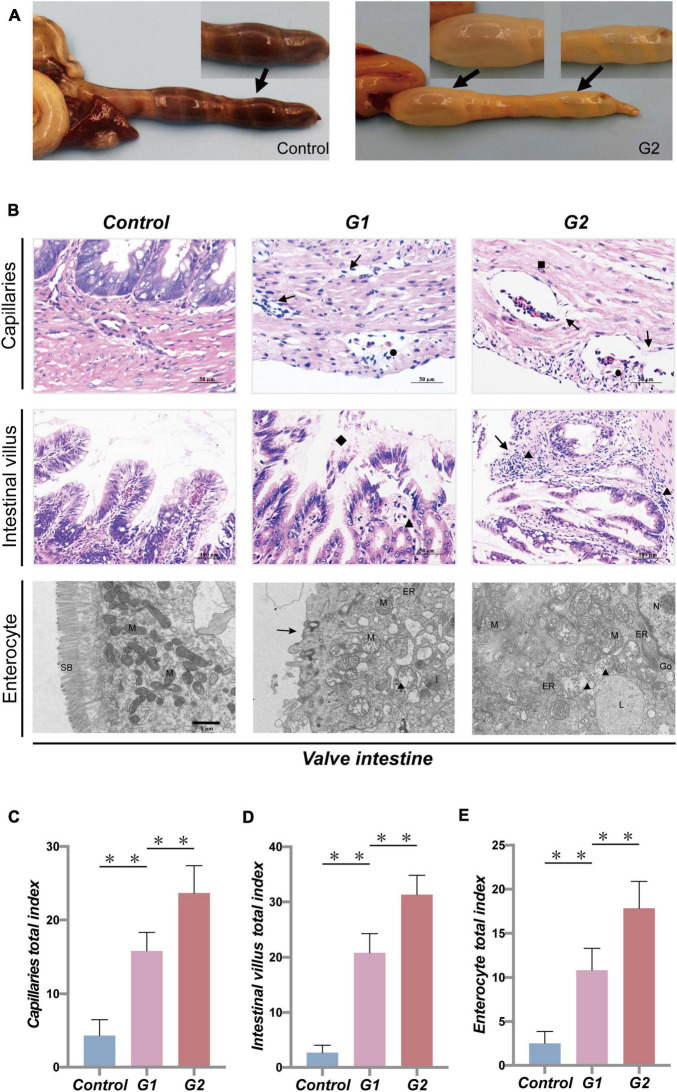
Systematic pathology of sturgeon gastrointestinal tract under heat stress. **(A)** Gastrointestinal lesions caused by heat stress (→, flatulence and no feces). **(B)** Histopathological changes in valve intestine induced by heat stress seen in the capillaries, intestinal villus (▲, inflammatory cell infiltration; →, necrotic cells; ■, edema; ●, congestion; ◆, loss of intestinal villi), and intestinal epithelial cell (N, nucleus; M, mitochondria; ER, endoplasmic reticulum; L, lysosome; ■, chromatin contracts; ▲, mitochondrial enlargement and rupture; →, microvilli shedding). **(C–E)** Overall health status (gross index) of sturgeon based on the histopathological damage in capillaries, intestinal villus, and intestinal epithelial cells, respectively. (**) Indicates extremely significant differences between groups (*p* < 0.01).

Anatomical results showed that the valve intestine in the sturgeons of group G2 had dilated due to substantial amounts of gas, despite being devoid of food, and the valve intestinal walls had high transparency (Figure 2A). To further explore the effect of heat stress on the gastrointestinal tract, pathological changes were evaluated. Pathological lesions occurred in the valve intestine and were most serious in the G2 group (Figures 2C,D). Mucosal epithelial cells showed marked signs of necrosis, and shedding cells were seen in the lumen of the valve intestine accompanied by a large amount of inflammatory cell infiltration (Figure 2B). The ultrastructural changes showed that heat stress had led to an enlargement of the mitochondria and endoplasmic reticulum, solidified chromatin, and blurring of the membrane boundary of the valvular intestinal epithelial cells (Figures 2B,E). The valve intestine of sturgeons in the elevated temperature group showed enteritis symptoms. No obvious lesions were present in the stomach or duodenum in any group, and no lesions were present in the valve intestinal of the control group.

### Heat Stress Disturbs the Intestinal Microbiota Diversity of Sturgeons

To further examine the relationship between intestinal damage and intestinal microbiota in sturgeons at high water temperatures, the compositions and changes of the bacterial and fungal microbiota of the intestinal feces were analyzed. Through optimizing sequencing results and statistical analysis, a total of 479,104 effective bacterial and 611,088 effective fungal DNA sequences were obtained from nine intestinal samples, each sample representing the intestinal feces of five fish. High-quality reads were clustered based on a >97% sequence identity into 45,785 bacterial and 56,847 fungal OTUs. Rarefaction curves showed that these OTUs represented sufficient coverage and accurately reflected the bacterial and fungal compositions (Supplementary Figures 1A,B). Alpha diversity was used to evaluate the diversity of bacteria and fungi in the intestines of sturgeons. Almost all bacterial and fungal diversity indexes showed upward trends as the water temperature increased (Table 3).

**TABLE 3:** Alpha diversity of intestinal bacteria and fungi of sturgeon after exposure to high water temperature for 12 days.

	Sample/Item	Sobs	Shannon	Simpson	Ace	Chao
Bacterial community	Control	192.67 ± 84.82	1.19 ± 0.39	0.52 ± 0.06	274.00 ± 29.74	228.54 ± 57.15
	G1	149.00 ± 19.80	1.24 ± 1.24	0.50 ± 0.18	173.89 ± 19.51	172.42 ± 17.80
	G2	292.33 ± 195.84	1.81 ± 0.78	0.32 ± 0.32	334.64 ± 194.42	324.86 ± 194.40
Fungi community	Control	33.00 ± 8.49	1.38 ± 0.08^a^	0.37 ± 0.01^a^	34.36 ± 7.94	33.17 ± 8.37
	G1	54.00 ± 36.12	2.40 ± 0.02^b^	0.15 ± 0.01^b^	54.68 ± 37.08	54.26 ± 36.48
	G2	57.00 ± 27.09	2.05 ± 0.70^ab^	0.24 ± 0.14^ab^	58.38 ± 27.13	57.20 ± 27.32

*Data are presented as mean ± SD (n = 9). Different lowercase superscript letters indicate significant differences between treatments (p < 0.05).*

### Explosive Increase of Thermophilic Microbiota Triggers Intestinal Microbiota Disorders

**FIGURE 3:**
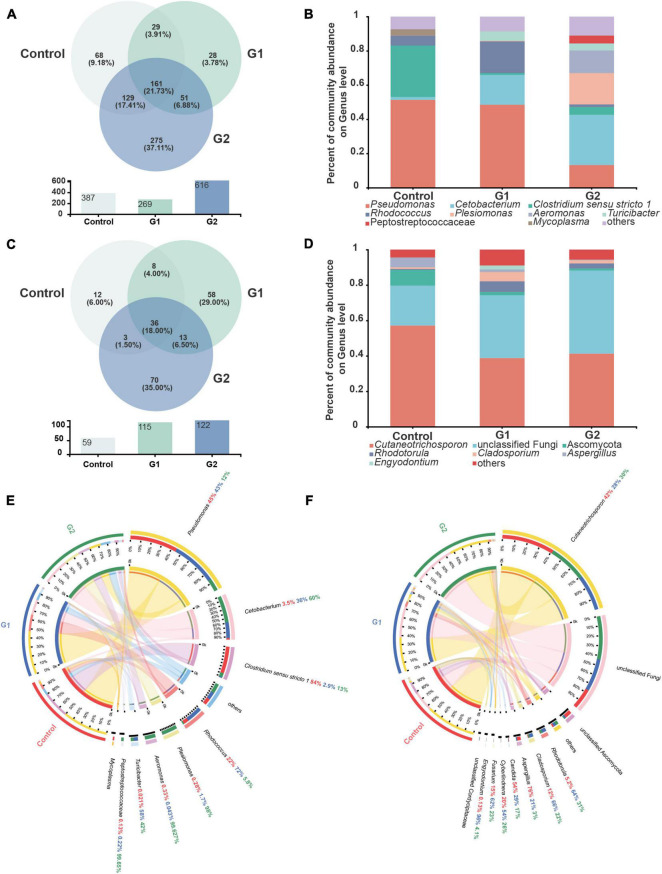
Composition of intestinal bacteria and fungi in sturgeon after exposure to high water temperatures for 12 days. **(A)** Venn diagram of unique and shared operational taxonomic units (OTUs) in bacterial microbiota. **(B)** Bar plot of the bacterial microbiota at the genus level. **(C)** Venn diagram of unique and shared OTUs in fungal microbiota. **(D)** Bar plot of the fungal microbiota at the genus level. **(E)** Intestinal bacterial composition at the genus level in different groups of sturgeon. **(F)** Intestinal fungal composition at the genus level in different groups of sturgeon.

The species, quantities, and proportions of intestinal microbiota were used to directly observe the differences in the microbiota of different treatment groups (Valdes et al., 2018; Aggeletopoulou et al., 2019). The Venn diagram illustrated that the number of OTUs of both bacteria and fungi was higher in the elevated temperature treatment groups than in the control group (Figures 3A,C). The microbiota composition analysis revealed that the bacterial microbiota of the control and heat treatment groups had significantly different dominant genus composition ratios. In the control group, *Pseudomonas* (51.36%) and *Clostridium sensu stricto* 1 (29.99%) were the dominant genera, whereas the proportion of dominant bacterial genus in the G2 group has changed significantly. The bacteria *Cetobacterium* (29.36%), *Plesiomonas* (18.24%), and *Aeromonas* (13.29%) all exhibited significant increases in abundance, while the abundances of *Pseudomonas* (13.28%) and *C. sensu stricto* 1 (4.58%) declined (Figure 3B). The increased diversity of fungi is reflected in the non-dominant genus, which increased significantly in the G2 group, reflected in the abundance of *Rhodotorula* (0.48 to 2.87%) and *Cladosporium* (0.94 to 1.79%). As the water temperatures rose, the abundances of the fungi *Cutaneotrichosporon* (57.17 to 43.34%), Ascomycota (8.89 to 1.16%), and *Aspergillus* (5.51 to 0.22%) were significantly reduced (Figure 3D).

The visual circle diagram was used to reflect the distribution ratios of each group of the dominant genus in the samples, as well as the distribution ratios of each dominant genus in separate groups. The top eight genera (others) in the dominant ratios were analyzed. The relative abundance values observed for each genus in the bacterial microbiota in the control group were *Pseudomonas* (45.00%), *Cetobacterium* (3.50%), *C. sensu stricto 1* (84.00%), *Rhodococcus* (22.00%), *Plesiomonas* (0.28%), *Aeromonas* (0.33%), *Turicibacter* (0.02%), and *Peptostreptococcaceae* (0.13%). The proportions of *Cetobacterium* (60.00%), *Plesiomonas* (98.00%), *Aeromonas* (99.63%), and *Turicibacter* (42.00%) increased significantly in G2. In contrast, the ratio of *Pseudomonas* (12.00%) and *C. sensu stricto 1* (13.00%) were significantly reduced by heat stress (Figure 3E). A quantitative analysis of the bacteria whose abundance had increased significantly showed that the average abundance value in the control, G1, and G2 were as follows: *Cetobacterium* 744, 8,483 and 14,201, respectively; *Plesiomonas shigelloides* 26, 150, and 8,332, respectively; and *Aeromonas veronii* 17, 1, and 6,081, respectively. The relative abundances of the fungal genera in the control group were *Cutaneotrichosporon* (42.00%), *Rhodotorula* (5.20%), *Cladosporium* (12.00%), *Aspergillus* (76.00%), *Candida* (54.00%), *Cyberlindnera* (20.00%), *Fusarium* (15.00%), and *Engyodontium* (0.13%). The proportions of *Aspergillus* and *Candida* decreased in G2, but the proportions of *Rhodotorula* and *Cladosporium* increased (Figure 3F).

**FIGURE 4:**
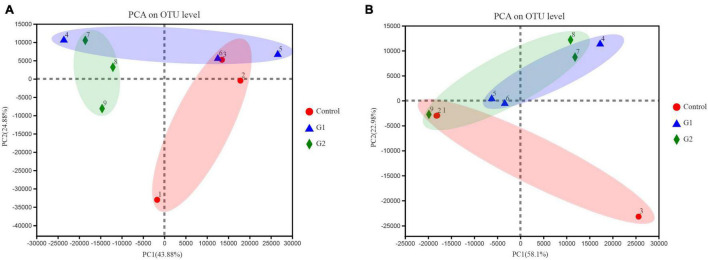
Principal coordinate analysis (PCoA) of microbiota differences among the three treatments. **(A)** PCoA of bacterial microbiota. **(B)** PCoA of fungal microbiota.

To better illustrate the change in genus abundances within the intestinal microbiota, the top 50 most abundant genera were selected to make a heat map. The horizontal abundance of the bacterial population between the G1 and control groups showed an intuitive dynamic change trend. *Mycoplasma* and *Brevinema* decreased significantly with increasing water temperature, while *Plesiomonas*, *Aeromonas*, *Faecalibacterium*, and *Turicibacter* gradually increased in abundance with increasing temperature (Supplementary Figure 2A). In the fungal microbiota, half of the population represented by *Alternaria* and *Talaromyces* increased in group G2 compared with the control and G1 groups, but *Xerochrysium*, *Penicillium*, *Xeromyces*, and *Aspergillus* all decreased in group G2 (Supplementary Figure 2B). Principal component analysis (PCA) was used to perform a simplified analysis of the data set, and it showed that heat stress caused a significant difference between the group control and the G2 in bacterial (Figure 4A) but in fungal microbiota, and the control and G2 groups had a sample overlap (Figure 4B).

### Heat Stress Suppresses the Digestive and Antioxidant Function of the Valve Intestine in Sturgeons

**FIGURE 5:**
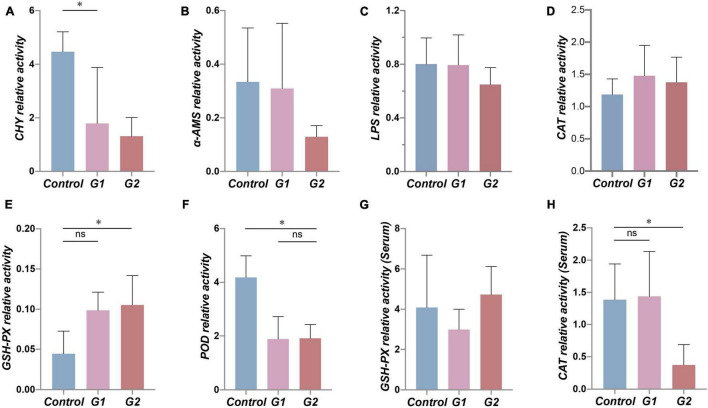
Antioxidant and digestive capacity in the valve intestine and plasma of different groups. **(A–F)** Assessment of CHY, α-AMS, LPS, CAT, GSH-PX, and POD enzyme activity in sturgeon valve intestine. **(G,H)** Assessment of CAT and GSH-PX enzyme activity in sturgeon plasma. (*) Indicates significant differences between groups (*p* < 0.05). CHY, chymotrypsin; α-AMS, alpha amylase; LPS, lipase; CAT, catalase; GSH-Px, glutathione peroxidase; POD, peroxidase.

To examine the functional changes in the valve intestine, the digestive ability and antioxidant activity were determined in the valve intestine and in the plasma by measuring CHY, α-AMS, LPS, CAT, POD, and GPH-PX. The activity of CHY, α-AMS, and LPS in the intestines and POD and CAT activity in the plasma decreased with increasing water temperature (Figures 5A–C,F,H). The CHY, POD, and CAT activities were significantly lower in the elevated temperature treatment groups G1 and G2 (Figures 5A,F,H: *p* < 0.05). The results suggested that the decreased activity of digestive and antioxidant enzymes in the valve intestine may be ascribed to heat stress.

### Reduced Thermal Tolerance and Repair Ability of Valve Intestine

**FIGURE 6:**
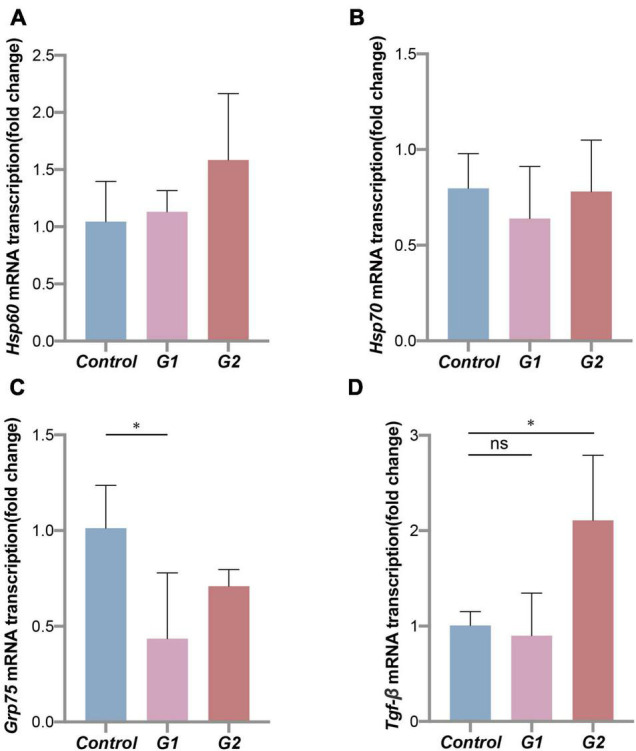
Thermal tolerance capacity of sturgeon in different groups. Expression of heat shock proteins **(A–C)** and transforming growth factor **(D)** mRNA in sturgeon from different groups. (*) Indicates significant differences between groups (*p* < 0.05).

The heat shock protein family genes *Hsp60*, *Hsp70*, and *Grp75* and transforming growth factor *Tgf-*β were used to assess the stress status of the valve intestinal tissue. The analysis showed that *Grp75* expression was significantly lower in the elevated heat treatments (G1) (Figure 6C) (*p* < 0.05). Of the remaining genes, *Hsp60* and *Tgf-*β showed the same trends, and *Tgf-*β transcription was significantly higher in the G2 group (Figures 6A,D). Most of these results indicated decreases in thermal tolerance and repair capacity of the valve intestine at elevated temperatures.

## Discussion

Heat stress reduces the availability of suitable habitats for cold-water fish in freshwater environments and impacts their physiological and metabolic processes (Larnier et al., 2010; Schram et al., 2013; Huang and Wang, 2018; Zhang H. et al., 2019), directly affecting the health and biological function of cold-water fish (Miegel et al., 2010; Larios Soriano et al., 2018). The normal function of the intestine requires the participation of a healthy intestinal microbiota (Larios Soriano et al., 2018). Previously, high water temperatures have been reported to affect the intestinal microbiota of *Salmo salar* more than their diet (Neuman et al., 2016). Within the microbiota, bacteria and fungi play important roles in promoting food digestion, nutrient absorption, and the immune system (Yap and Marino, 2018). Under suitable water temperatures, beneficial microbiota can flourish, and high relative abundances of beneficial microbes can be maintained, helping to maintain the host’s metabolic capacity and health (Dehler et al., 2017). In this study, the number of species and abundance of thermophilic bacteria and fungi were elevated in sturgeons exposed to high water temperatures, which was consistent with the results of the study by Soriano et al. on Yellowtail Kingfish in 2018, which showed that increases in ambient temperature increased the diversity of intestinal microbiota. The bacterial PCA in this study showed that there was a significant difference in composition between the control and G2 species. However, the composition of fungi of samples 1 and 2 was like that of G2, which may be due to the individual differences of sturgeons under heat stress and the low sensitivity of most fungi to temperature increase. This study saw an explosive growth of thermophilic bacteria and the severe decline of psychrophilic bacteria during heat stress, which significantly changed the composition of the microbiota.

Previous studies reported that elevated temperatures had led to a significant increase in abundance of certain bacteria, such as *Plesiomonas* and *Aeromonas*, which had been found to have some degree of pathogenicity in *Hypophthalmichthys molitrix* and *Lateolabrax maculatus* (Behera et al., 2018; Wang et al., 2021). In the present study, the abundance and proportions of the thermophilic bacteria *Plesiomonas* and *Aeromonas* increased after heat stress. Studies have shown that outbreaks of pathogenic bacteria caused by high water temperatures can invade the blood from the intestine and lead to severe enteritis (Kanai et al., 2006; Chen et al., 2020). In this study, the relative abundance of *Plesiomonas*, which is a common aquatic pathogen that is speculated to be responsible for enteritis in sturgeons, was higher in G2 (Behera et al., 2018; Gong et al., 2019; Zhang M. et al., 2019). However, the abundance of *Plesiomonas* only increased significantly in two samples of the G2 group, which may be due to host differences and the low basic quantification of *Plesiomonas*.

Aiming at the phenomena occurring in the valve intestine, Huang et al. (2020) speculated that *Clostridium* was the main cause of excess gas production. Based on the changes in genera compositions and abundances in the high-temperature group (G2), *Cetobacterium* and *Aeromonas* were suspected to be the main causes of valve intestine flatulence in this study (Wiegel et al., 2006; Ma et al., 2009). Fungi with increased abundance after heat stress, such as *Rhodotorula*, are not pathogenic in research reports (Raggi et al., 2014; Boguslawska-Was et al., 2019), and *Cutaneotrichosporon* has no pathogenicity studies. Therefore, we can deduce that it was the large proliferation of gas-producing bacteria caused by temperature elevation that induced the flatulence, decreased food intake, and no feces in the valve intestine of sturgeons.

In this study, no feces was present in the intestines of the high-temperature group, and a large number of pathogenic bacteria may attack the host’s intestinal mucosa and cause serious damage to the intestinal tissue (Desai et al., 2016). The histopathological results of the sturgeon intestine in group G2 were similar to the enteritis symptoms reported by Huang et al. (2020), so it was speculated that heat stress caused sturgeon enteritis. Research has shown that environmental temperatures may influence physiological metabolic processes in fish by driving dramatic changes in the composition and abundance of intestinal microbiota (Kuz’mina et al., 2012, 2019) and may inhibit the secretion of digestive enzymes and damage the structure of digestive enzymes (Ahmadifar et al., 2020). Clostridiaceae has been recognized as a beneficial bacteria that is involved in the breakdown of carbohydrates and proteins (Kim et al., 2008; Wuest et al., 2011), but the high water temperatures in this study inhibited their growth and led to a significant decrease in *C. sensu stricto* 1 abundance. Studies have indicated that Trichosporonaceae promotes the digestion and absorption capacities of hosts by secreting proteinases (Aliyu et al., 2020), but in this study, the abundance of *Cutaneotrichosporon* in the intestinal microbiota gradually decreased under high water temperatures, although a high metabolic rate was maintained. The transport and repair ability of proteins was reduced, and the proliferation of intestinal epithelial cells and vascular endothelial cells was inhibited.

Overall, the feed intake, the ability of digestion, and absorption of sturgeons were significantly inhibited in sturgeons, which was attributed to the damage of the intestinal structure, heat tolerance reduction, and reduced repair ability. Consequently, the sturgeons’ energy supply was blocked, and microbial consumption was disordered, which inevitably posed a threat to the growth and survival of wild and cultured sturgeons. The absence or proliferation of intestinal microbiota in fish may lead to impaired physiological functions, such as intestinal epithelial cell dysfunction, compromised nutrient absorption, and metabolism (Wang et al., 2018). Therefore, a series of preventive measures will be indispensable in the protection and breeding of sturgeons. First and foremost, stable habitats with suitable water temperatures especially in summer play a vital role in the conservation of wild endangered sturgeons and breeding of farmed ones. Regular monitoring of both water temperature and the sturgeons’ behavior is needed during farm management. Water cooling should be undertaken as soon as the water temperature in ponds rises dramatically to protect physiological functions and the maintenance of intestinal microbiota. In summer, the regular addition of endogenous probiotics such as *Bacillus* spp. and prebiotics to the diet or aquaculture water may protect the sturgeon intestines from the negative effects of heat stress (Geraylou et al., 2013). Natural extracts including resveratrol and arabinoxylan-oligosaccharides can also be added to the diet to enhance the anti-oxidation and antibacterial abilities of the sturgeon intestines and improve the growth performance (Geraylou et al., 2012; Liu et al., 2018; Zheng et al., 2018; Farsani et al., 2021). On the whole, effective and timely precaution can be essential to the resistance to heat stress for the protection and breeding of sturgeons and other cold-water fishes.

## Conclusion

This study demonstrated that heat stress triggered the disturbance of the intestinal microbiota in *A. baerii* ♀ × *A. schrenckii* ♂ hybrid F1. The explosive increase of thermophilic microbiota and pathogenic bacteria genera including *Plesiomonas*, *Cetobacterium*, and *Aeromonas* may be associated with the development of enteritis. This was followed by the serious inhibition of intestinal digestion and antioxidant function. Simultaneously, heat stress reduced the thermal tolerance and weakened the repair ability of the valve intestine in sturgeons. The present work will be helpful for strategies making to enhance the resistance to thermal stress for wild and cultured sturgeons.

## Data Availability Statement

The datasets presented in this study can be found in online repositories. The names of the repository/repositories and accession number(s) can be found in the article/Supplementary Material.

## Ethics Statement

The animal study was reviewed and approved by Animal Care and Use Committee of Sichuan Agricultural University Sichuan Agricultural University.

## Author Contributions

SY, CZ, and WX contributed to the data detection, conception, and design of the study. DL organized the database and data analysis. YF performed the statistical analysis. SY and CZ wrote the draft of the manuscript. JW, WL, and XD wrote sections of the manuscript. ZD and XH were responsible for reviewing and submitting articles. All authors contributed to manuscript revision and read and approved the submitted version.

## Conflict of Interest

The authors declare that the research was conducted in the absence of any commercial or financial relationships that could be construed as a potential conflict of interest.

## Publisher’s Note

All claims expressed in this article are solely those of the authors and do not necessarily represent those of their affiliated organizations, or those of the publisher, the editors and the reviewers. Any product that may be evaluated in this article, or claim that may be made by its manufacturer, is not guaranteed or endorsed by the publisher.

## Footnotes

1
http://drive5.com/usearch/manual/dereplication.html

2
http://drive5.com/usearch/manual/singletons.html

3
https://www.mothur.org/wiki/Download_mothur

## Supplementary Material

Supplementary Figure 1 Dilution curve of intestinal bacteria and fungi in sturgeon after exposure to high water temperatures for 12 days. **(A,B)** Represent dilution curves of bacteria and fungi, respectively.

Supplementary Figure 2 Heatmap of the bacterial and fungal microbiota at the genus level. **(A,B)** Represent heatmap of bacteria and fungi, respectively.
