# Supplementary figures and images for: Heat Stress Decreases Intestinal Physiological Function and Facilitates the Proliferation of Harmful Intestinal Microbiota in Sturgeons
Source: Front Microbiol. 2022 Mar 7;13:755369. doi: 10.3389/fmicb.2022.755369 (PMC8959899; doi:10.3389/fmicb.2022.755369)

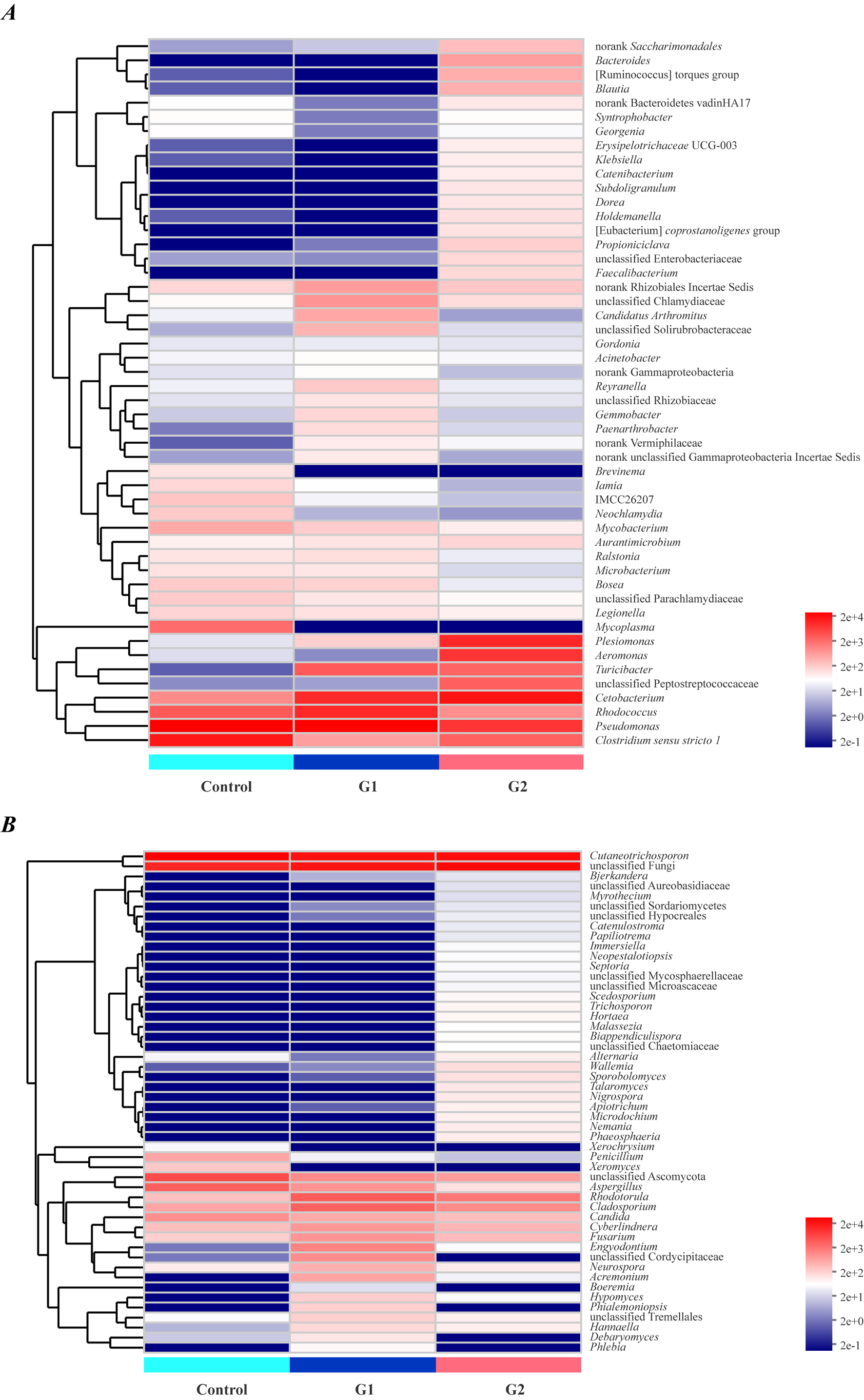

Supplement: Supplementary Figure 1 — Dilution curve of intestinal bacteria and fungi in sturgeon after exposure to high water temperatures for 12 days. (A,B) Represent dilution curves of bacteria and fungi, respectively. [file Image_1.TIF]

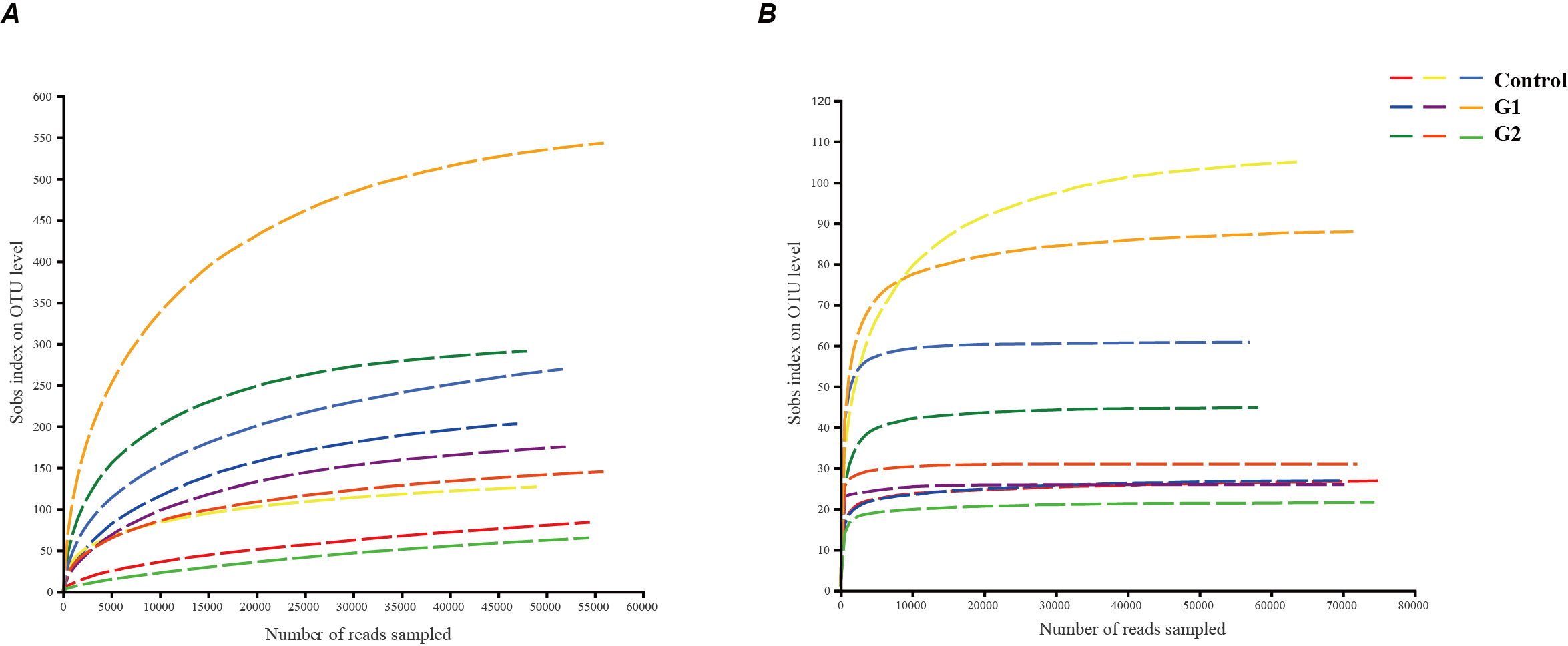

Supplement: Supplementary Figure 2 — Heatmap of the bacterial and fungal microbiota at the genus level. (A,B) Represent heatmap of bacteria and fungi, respectively. [file Image_2.JPEG]
