# Supplementary material for: Heat Stress Decreases Intestinal Physiological Function and Facilitates the Proliferation of Harmful Intestinal Microbiota in Sturgeons
Source: Front Microbiol. 2022 Mar 7;13:755369. doi: 10.3389/fmicb.2022.755369 (PMC8959899; doi:10.3389/fmicb.2022.755369)
Supplement: Supplementary file 3 [file Table_1.docx]

**Supplementary Table 1.** Microbial diversity analysis database and software.

| **Analysis software/database** | **Version number** | **Analyze software/database usage** |
| --- | --- | --- |
| Flash | 1.2.11 | Pair-end double-ended sequence splicing |
| Qiime | 1.9.1 | Taxonomic abundance table, Beta diversity distance calculation |
| Uparse | 7.0.1090 | OTU clustering |
| RDP Classifier | 2.11 | Sequence classification annotation |
| Usearch | 7 | OTU statistics |
| Mothur | 1.30.2 | Alpha diversity analysis |
| SILVA | 132 | rRNA database |
| UNITE | 8 | Fungal ITS database |
| RDP | 11.5 | rRNA database |
| GreenGenes | 135 | rRNA database |
| FunGene | 9.6 | Functional gene database |
| MaarjAM | 81 | Fungal 18S rRNA database |
| MAFFT | 7.2 | Multiple sequence alignment |
| Fastp | 0.19.6 | Quality control |
